# Induction of Apoptosis by Alcoholic Extract of Combination *Verbascum thapsus* and *Ginger officinale* on Iranian Isolate of *Trichomonas vaginalis*

**Published:** 2018

**Authors:** Zohreh FAKHRIEH-KASHAN, Mohsen ARBABI, Mahdi DELAVARI, Mahdi MOHEBALI, Hossein HOOSHYAR

**Affiliations:** 1. Dept. of Medical Parasitology and Mycology, School of Medicine, Kashan University of Medical Sciences, Kashan, Iran; 2. Dept. of Medical Parasitology and Mycology, School of Public Health, Tehran University of Medical Sciences, Tehran, Iran

**Keywords:** *Trichomonas vaginalis*, Alcoholic extract, *Verbascum thapsus*, *Ginger officinale*, In vitro

## Abstract

**Background:**

The protozoan *Trichomonas vaginalis* is a sexually transmitted disease (STD). Metronidazole is a chosen drug for the treatment. This study evaluated the anti trichomonal activity of alcoholic extracts of combination *Verbascum thapsus* and *Ginger officinale.*

**Methods:**

This experimental study was conducted in the Parasitology Laboratory, Kashan University of Medical Sciences, Kashan, Iran in 2015, on 23 women with suspected trichomoniasis referring to Kashan clinical centers. Medium TYI-S-33 was used for culture of three *T. vaginalis* isolates. Different concentrations (25, 50, 100, 200, 400, 800 μg/ml) of *V. thapsus* and *G. officinale* ethanol extract added to *Trichomonas* trophozoites in 48-well plates and metronidazole considered as positive control and the negative control was TYI-S33 containing *Trichomonas* trophozoites without any drug. In all of mentioned groups, trophozoites number counted 12, 24, 48 h after culture. Results were analyzed using ANOVA statistical test, to evaluate the toxicity of extract, measured by MTT assay. Induced apoptosis of *T. vaginalis* after treatment with different concentrations of extract was determined by Flow Cytometry.

**Results:**

IC50 of alcoholic extract of combination *V. thapsus* and *G. officinale* and metronidazole after 24h was 73.80 μg/ml and 0.0326 μg/ml, respectively. The toxicity percentage of 25–800 μg/ml concentrations of this combination were between 0.2–1.98. In different concentrations of extract (25,50,100,200 and 400 μg/ml) apoptosis percent after 48h was 18.97 to 77.19 and necrosis percent was calculated 1.35, 3.18, 3.10, 1.16 and 4.09, respectively.

**Conclusion:**

Alcoholic extract of combination *V. thapsus* and *G. officinale* induces programmed death in *T. vaginalis*. Due to no toxicity on macrophages, it can be examined in vivo studies.

## Introduction

*Trichomonas vaginalis* is a protozoan parasite that causes human trichomoniasis, a very sexually transmitted disease (STD) with a significant incidence worldwide. This flagellated protozoan parasite can infect the urinary tract-genital in women and men (1). Symptomatic women usually have common sites of infection include the vagina, urethra and endocervix and clinical features include vaginal discharge, dysuria, itching, vulvar irritation and abdominal pain, *T. vaginalis* in patients with AIDS associated with inflammation and cervical cancer (2, 3).

The prevalence rate of trichomoniasis in Iran is 2 to 8% and may be up to 30% in high-risk populations (4). In recent years, metronidazole is used in the treatment of this infection as the most effective drug. Some reports of potential carcinogenic and teratogenic effects on the fetus and the incidence of drug resistance have been confirmed (5, 6). Many attempts have been made for evaluation of the effects of plants on the *T. vaginalis* (7, 8). *Verbascum thapsus* belongs to the family of Scorphulariaceae seen throughout the world. Compounds of this plant reduce cyclooxygenase activity and other bioactive substances including phenyl ethanol, glycosoaminoglycans, glycosides, saponins, and it is antiseptic and anti-inflammatory properties and is known for its skin wound healing. In addition, the plant is used to treat diarrhea and urogenital system infection (9, 10).

*Ginger* belongs to the family of the Zingiberaceae, and contains flavonoids, saponins, tannins and alkaloids with powerful antioxidant properties. Ginger is used to treat nausea, vomiting, arthritis, rheumatoid arthritis and osteomyelitis applications. This plant has anti-bacterial and anti-viral effects (11). With the prevalence of this parasite and the effects of herbal medicines on this disease, the present study aimed to determine the effectiveness of alcoholic extract of combination *V. thapsus* and *Ginger officinale* compared with the metronidazole in vitro.

## Materials and Methods

### Parasite Culture

This experimental study was conducted in Parasitology Laboratry, Kashan University of Medical Sciences, Kashan, Iran 2015 on women with suspected trichomoniasis referred to health centers based on clinical examination and microscopic examination of wet vaginal secretions of infected persons. From 23 samples were infected by *T. vaginalis* three isolates were selected for study. The parasites were axenically grown in standard TYI-S33 (trypticase-yeast extract-maltose) medium (pH:6.8) supplemented with 10% FCS, vitamin mixture and 100U/ml penicillin and 100 μg/mL streptomycin mixture at 37 °C.

### Preparation of Plant Extracts

*V. thapsus* and *G. officinale* plants were obtained from the market and were approved by agricultural experts and then extracted according to the standards of British pharmacology, using the Percolation method (13).

### Anti-trichomonal assay

Trophozoites were cultured in TYM-S-33 media in 48-well plates (5×10^4^cell/well) as triplicate, different concentrations (25,50,100,200,400,800μg/ml) of combination *V. thapsus* and *G. officinale* ethanol extract and metronidazole (0.025,0.05,0.1,0.2,0.4 μg/ml) were added to each well individually. Metronidazole was used in injected form that made in manufacturing companies of Tabriz in Iran at a concentration of 0.5%. The number of parasites in each well plate was counted after 12, 24, and 48 h by trypan blue staining. In the negative control group, trophozoites were cultured in TYI-S33 without any drug. After counting of parasites, IC50 was determined by Graph Pad prism5 and growth inhibition percentage was calculated using the following formula:

A−B/A×100

A=Average number of trophozoites in control group. B=Average number of trophozoites in test group.

The present study was done according to the local ethics review committee of Kashan University of Medical Sciences that approved this work.

### Flow Cytometry Analysis of Cell Death

Treated parasites were collected after 48h and centrifuged at 2000 rpm for 5 min. Then supernatant was removed, and 500μl binding buffer, 5μl Annexin-V and 5μl Propidium iodide (PI) were added to the residue. The samples incubated at laboratory temperature and dark condition for 5min. Absorbed color intensity in cells was observed by flow cytometry (2005 by Partec GmbH Munster, Germany) and the results were analyzed by FlowJo software, and the rate of apoptosis was determined.

### Toxicity evaluation of combination *V. Thapsus* and *G. officinale* ethanol extract

Peritoneal macrophages obtained from mice and 10^5^ cells/well macrophages were cultivated on each well of the 96-well plates and treated with different concentrations of the combination *V. thapsus* and *G. officinale* ethanol extract for 12, 24 and 48h at 37 °C. After this time, 20 μl MTT reagents (5 mg/ml, pH 7.4) in fresh TYI-S-33 culture medium were added to each sample. Then the plates were incubated for 3–5h at 37 °C under 5% CO2. After this time, the supernatant was removed from wells and 100 μl of DMSO was added to each well. After 15 min, absorption (OD) of each well was read at 570 nm by an ELISA reader. The amount of killed macrophages was determined based on the optical absorbance in test and control groups using the following formula (14).

Killed macrophage (%)=1−(AT−AB)/(AC−AB)×100.

AB is the OD of the blank well, AC is the OD of the untreated samples and AT is the OD of treated samples.

## Results

The effect of this combination was measured at different concentrations (from 25 to 800 μg/ml) and at 12, 24 and 48h. There were no any motile and alive trophozoites at 800μg/ml concentration of extract after 48 h. IC50 for extract and metronidazole was calculated 73.8 μg/ml and 0.0326 μg/ml respectively (Table 1).

**Table 1:** Comparison of the effects of different concentrations of the *V. thapsus* and *G. officinale* ethanol extract on *T. vaginalis*, 12 h, 24 h and 48 h after exposure

***Concentrations (μg/ml)***	***T. vaginalis trophozoite (× 10^4^), Mean±SD***		
	**12 h**	**24 h**	**48 h**
25	40.7 ± 0.57	35.6±6.02	31.7±3.78
50	35.33± 4.5	29.7± 1.52	26.3±5.50
100	29.7± 0.57	26.3±2.51	20.7± 7.02
200	25.3 ± 4.5	20.3±0.57	16.7±1.53
400	17±3	10.7±1.15	4.7 ± 0.57
800	0.67±0.57	0.57±0.33	0
0.25MZ	20±0.6	8±1.2	0
0.05MZ	12±0.4	2±0.3	0
0.1MZ	8±0.3	0	0
0.2MZ	6±0.5	0	0
0.4MZ	2±0.7	0	0
Negative control	38.33±2.51	53.66±3.51	88.3±6.5
Statistical comparison of groups	***P*<0.05**		

### MTT assay

The toxicity of the combination *V. thapsus* and *G. officinale* ethanol extract at concentrations (25, 50, 100, 200, 400, 800 μg/ml) in three times (12, 24, 48 h) were obtained using the MTT assay (Table 2). After 12h the combination *V. thapsus* and *G. officinale* ethanol extract in 25–800 μg/ml concentrations the fatality percent was between 0.2–0.50. It was between 0.51–1 and 1.01–1.98 after 24 and 48h respectively. Toxicity of the combination *V. thapsus* and the *G. officinale* ethanol extract at the highest concentration (800 μg/ml) and time (48 h) was 1.98% in macrophage cells (Table 2).

**Table 2:** Comparison of the fatality percent alcoholic extract of combination *V. thapsus* and *G. officinale* on macrophages isolated from mice, 12 h, 24 h and 48 h after exposure

***Concentrations*** (μg/ml)	***Percentage of toxicity (%)***		
	**12h**	**24h**	**48h**
25	0.20	0.51	1.01
50	0.25	0.66	1.12
100	0.33	0.78	1.21
200	0.38	0.87	1.31
400	0.45	0.99	1.46
800	0.50	1	1.98

### Flow Cytometry Analysis

**Fig. 1:**
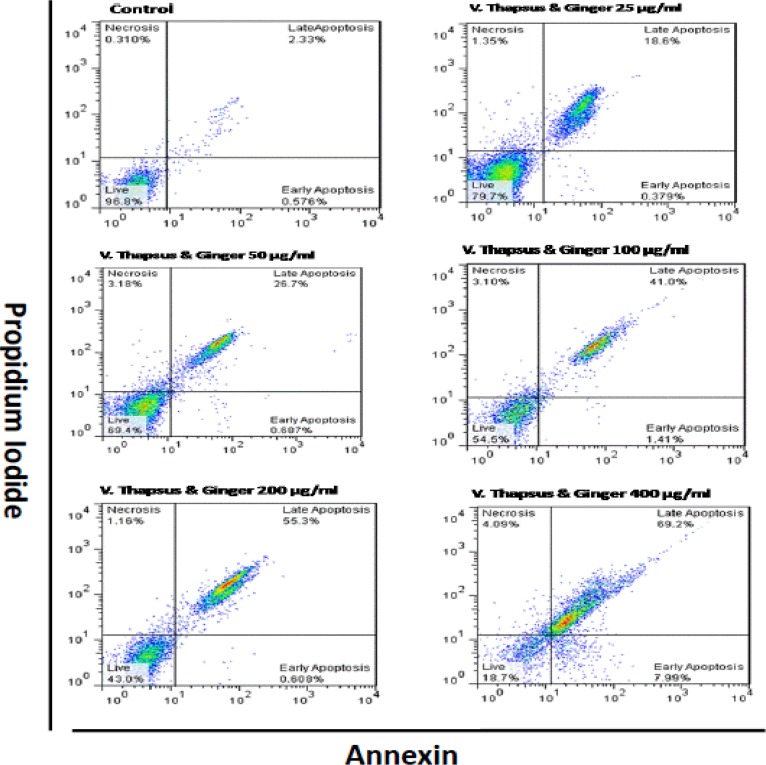
Flow cytometry of results showed the extract had considerable induced apoptosis and also low necrotic effects on *T. vaginalis* trophozoites

An alcoholic extract of combination *V. thapsus* and *G. officinale* induce apoptosis in trophozoites. Necrotic and apoptotic effects of alcoholic extract of combination *V. thapsus* and *G. officinale* on the trophozoites have been shown in (Fig. 1). Induced apoptosis (Early and Late) after 48 h in 25,50,100,200 and 400 μg/ml concentrations of the extract was 18.97 to 77.19 and necrosis was calculated 1.35%, 3.18%, 3.10%, 1.16% and 4.09% respectively (Fig. 1). Because the number of parasites after 48h at concentrations 800 is zero, Flow cytometry analysis was not done in this concentration.

## Discussion

The number of the parasites at 25 and 100 μg/ml concentrations of extract reduced after 12 and 24h respectively, and IC50 was calculated 73.80 μg/ml after 24h. In this study, IC50 for metronidazole was calculated 0.0326 μg/ml, that is in similar to results of recently published data (IC50:0.0326) (14). The alcoholic and hydroalcoholic extract of Pelargonium has anti-Trichomonas effect; the anti-*Trichomonas* properties of alcoholic extract or more than its aqueous extract and the IC50 of the aqueous and alcoholic extracts of *Pelargonium* after 24h were 54.67, 27.63μg/ml respectively. In another study, the alcoholic extracts of *Allium cepa*, *Oliveria decumbens Vent,* and *Muscari neglectum* had an inhibitory effect on in vitro growth of *T. vaginalis*. The IC50 rate was calculated 101.8μg/ml for *Olivera documents Vent*, 572.3μg/ml for *Allium cepa* and 329.4μg/ml for *Muscari neglectum* after 24 h (15, 16). Lavender oil in all concentrations has an inhibitory effect on the *T. vaginalis* (17). Extract, *Papaya* and *Cocos* have powerful anti-trichomonal effect and extract *Bocconia frutescens*, *Geranium mexicanum*, *Lygodium venustum* have effective anti-trichomonal activity (5). Flavonoids, Saponins, Tannins, Terpenoids, Glycoside, Carbohydrates, Proteins, Fats, and oils that have effective anti-*Giardia lamblia* (18), anti-*Trichomonas galina* (19), anti-*T.vaginalis* (20), anti-*Acanthamoeba castellani* (21) and anti *L. infantum* activity (22). *Ginger* had higher anti-*Schistosoma mansoni*, *Angiostrongylus cantonensis* and *Anisakis* larvae activity (23, 24).

According to the results of flow cytometry in the present study, in the control group, 96.8% of cell was alive and percentages of necrosis, early apoptosis, and late apoptosis were 0.310%, 0.576% and 2.33% respectively. Induced apoptosis (Early and Late) by 25, 50, 100, 200 and 400 μg/ml of extract was 18.97%, 27.3%, 42.41%, 55.9% and 77.19%, respectively.

The results of flow cytometry showed that combination *V. thapsus* and *G. officinale* ethanol extract after 48h induction of apoptosis. Treatment of *T. vaginalis* with metronidazole does not lead to necrosis and causes cell death by apoptosis (25). The effect of anti-*T. vaginalis Sapindus* saponins were examined and the results showed the extract induced apoptosis (26). IC50 of *V. thapsus* ethanol extract after 24h was 39.17 μg/ml and the effect of the *Verba scumthapsus* ethanol extract on induced apoptosis in *T. vaginalis* was determined by Flow Cytometry and toxicity of *V. thapsus* alcoholic extract on mice macrophages was observed between 0.17–0.25 after 12 h and they were between 0.25–0.42 and 0.45–0.95 after 24 and 48h respectively (27). Toxicity of alcoholic extract of combination *V. thapsus* and *G. officinale* at the highest concentration (800μg/ml) and time (48h) was 1.98% in macrophage cells. Therefore, these extract as no toxicity in BALB/c mice peritoneal macrophages. Alcohol extract, Ginger was evaluated on liver cells and was IC50:2500μg/ml and without any toxic effect (28).

## Conclusion

Alcoholic extract of combination *V. thapsus* and *G. officinale* induces programmed death in *T. vaginalis*. No toxicity on infected macrophages was observed. More comprehensive studies are needed to survey anti-*Trichomonas* activity of alcoholic extract of combination *V. thapsus* and *G. officinale* in vivo conditions.
